# A distributed geometric rewiring model

**DOI:** 10.1038/s41598-024-61695-y

**Published:** 2024-05-15

**Authors:** Magali Alexander Lopez-Chavira, Daniela Aguirre-Guerrero, Ricardo Marcelín-Jiménez, Luis Alberto Vásquez-Toledo, Roberto Bernal-Jaquez

**Affiliations:** 1 El Colegio de México, Programa Interdisciplinario en Ciencia de Datos, 14110 Tlalpan, Mexico; 2 Department of Applied Mathematics and Systems, Universidad Autónoma Metropolitana, 05348 Cuajimalpa, Mexico; 3 Department of Electrical Engineering, Universidad Autónoma Metropolitana, 09340 Iztapalapa, Mexico

**Keywords:** Complex networks, Applied mathematics, Computer science

## Abstract

We propose a distributed rewiring model which starts with a planar graph embedded into the Euclidean space and then behaves as a distributed system, where each node is provided with a set of dynamic links. The proposed rewiring evolves through cycles, where nodes explore the network to identify possible shortcuts and rewire their dynamic links. The rewiring decisions are subject to Euclidean and geodesic distance constrains. The emerging networks were assessed through topological and robustness analyses. We found that the networks display a variety of characteristics observed in complex networks encompassing phenomena such as preferential attachment, the distinctive traits of small-world networks, the presence of community structures, and robustness against degradation process. We consider that our proposal can be applied in the design of those self-managed systems in which there is a limitation on communication resources that can be represented by the Euclidean distance and, however, the components themselves can deploy strategies to optimize the transport of information and develop tolerance before contingencies.

## Introduction

Nowadays, the analysis and comprehension of complex systems have emerged as a central topic in various fields. From social networks to biological systems and infrastructure networks, the interconnection of elements within these systems has encouraged the development of various network models aimed at both unraveling their underlying structures and improving their robustness and performance, either by adding more resources to the network^1^ or by optimizing its resources^2^. Among these models, those based on rewiring strategies particularly capture our attention due to their crucial role in understanding how networks evolve, adapt, and function in various domains.

The most common approach that can be identified is where the entire system is considered as a single entity, and therefore rewiring decisions are made in a centralized manner or with full knowledge of the underlying graph. The classic example of this kind of rewiring process is the Watts-Strogatz model^2^, in which there is a centralized algorithm that modifies the network. It starts with a regular lattice graph (a ring graph with *n* vertices, each of them connected to *k*/2 neighbors on each side) and then rewires some edges randomly. This process allows the graph to evolve from a highly clustered, regular graph to a small-world graph. Similarly to the Watt-Strogatz model, most of the research on the subject focuses on proposing and analyzing rewiring strategies to understand their implications in the topological properties^3,4^ and the dynamic processes experienced by the resulting networks^5,6^. Kleinberg’s model^21^ follows both approaches, based on Milgram’s experiment^7^ to create small-world graphs with the property of being *“navigable”*. Navigability on networks refers to having very limited search times in the network, of the order of $O(log(n))^2$, where *n* is the number of vertices, this property facilitates efficient searching within the network, even as it scales up, by allowing nodes to discover the shortest paths across the network. On the other hand, there are studies that analyze the implication of network rewiring on specific domains. In neuroscience, for example, rewiring can represent changes in neural connections. Understanding how the brain reconnects itself in response to experiences such as learning, injuries, or diseases is a critical research area^8,9^. In social networks, rewiring can represent changes in relationships or interactions between individuals and communities. Studying how social networks evolve through the settlement of new connections can provide insights into social dynamics^10^. Granovetter’s work^11^, for instance, shows that weak ties play a critical role in spreading information and offering links between the otherwise isolated communities. Also, these links provide the initial exploration ways to discover potential shortcuts. Meanwhile, in infrastructure networks, these ideas can be used for resource optimization and robustness improvement^12^.

Unlike their centralized counterparts, distributed models do not rely on a single central entity to manage changes. Instead, decisions are made locally at each node, leading to the emergence of distinct global properties and topologies. We found this distributed approach in Peer-to-Peer network protocols (P2P) like Chord^13^, where nodes are regarded as being part of an initial ring-like graph. Each node decides its later connections in order to reach the different regions of the network. This reorganization is accomplished under a fair load distribution. Also, it is worth mentioning the Symphony protocol^14^, which is inspired by the Watts-Strogatz model, and is deployed over a ring-like network to achieve structural properties similar to those of small-world networks. On the other hand, none of these works considers neither the restrictions on the length of the links, nor the embedding of the graph within a metric space.

Working in complex networks cannot only be approached from a theoretical perspective. It is also important to use experimental tools such as discrete event simulation and agent-based models, with which it is possible to evaluate the distributed nature of self-organization, as is the focus of our proposal. Let us recall that complex systems are very sensitive to initial conditions. We are interested in studying the impact of conditions such as reconnection rules, restrictions on the range of links, the initial neighborhood, as well as the behavior of the system under different types of degradation processes. In this sense, our approach is related to the work of Namatame^15^ and D’angelo^16^. To our best knowledge the rewiring models that show some resemblance with our proposal are those observed in the work of Colman^4^ and the work of Rentzeperis and van Leeuwen^8^, respectively. In the case of Colman, even though there are rules that take into account local aspects and partial information for rewiring, they are still done from a centralized perspective; in this case, network restrictions are not taken into account. Indeed, the algorithm considers that each node knows the identity of every node in the system, which can be hardly assumed from a decentralized perspective. In the case of Rentzeperis and van Leeuwen, the rewiring is also carried out in steps and for each step, those nodes in which there could be the most intense activity are chosen for rewiring. Nevertheless, the evaluation of this characteristic and the choices are also carried out centrally.

In this paper, we continue with a previous work on distributed rewiring for complex networking^17^ and, we now propose a distributed geometric rewiring model that works as follows. It starts with a planar graph as initial topology, which is embedded into the Euclidean space, and then behaves as a distributed system, where nodes are agents able to exchange information through a geometric routing algorithm, called Compass Routing^18^. Each node in the initial planar graph is provided with a set of additional links, called dynamic links, that can be rewired under a Euclidean distance constraint, which is given as initial condition. Meanwhile, links belonging to the initial planar graph remain fixed. The rewiring process is executed by cycles, where nodes send tracer packets to explore the network and to identify potential shortcuts. Based on this information and the Euclidean distance constraint on dynamic links, nodes follow a simple rewiring rule to establish shortcuts that allow them to reduce the geodesic distance between nodes. Note that the rewiring mechanism works with 2 complementary measures that evaluate the distance between nodes: Firstly, the Euclidean distance, refers to the space where the initial planar graph is embedded and can be assessed as the distance between 2 points in this metric space. On the other hand, the geodesic distance refers to the length of the shortest path between 2 nodes of the appointed graph. The Euclidean distance between nodes remain fixed along the rewiring process. In contrast, geodesic distance between nodes evolves, and indeed is shortened, as result of the rewiring process.

An important distinction of this research work from our previous work^17^ is that we have redefined the rewiring rules to clarify that they are driven by two distance measures: the Euclidean and geodesic. In addition, we not only analyze the topological properties of the resulting networks, but also we analyze their entropy, community structure and robustness against failures and attacks scenarios. Our findings show that the resulting networks exhibit various combinations of structural and functional properties similar to those observed in complex networks, such as the preferential attachment phenomenon, small-world characteristics, community structure, robustness against random failures, etc. We consider that our proposal can be applied in the study of those self-managed systems in which there may be a limitation of communication resources and, however, the components themselves can deploy strategies to optimize the transport of information and tolerance before contingencies. Furthermore, our research provides insights into the evolution and robustness of networks where the cost of link rewiring depends on a distance metric different from the geodesic distance, such as in telecommunication networks, where this cost depends on the Euclidean distance.

In the literature on complex networks, rewiring models are notable for their unique focus on modifying existing connections to optimize or alter the network structure. The present work proposes a methodology to selectively adjust the connections between nodes in a strategic and methodical manner, its main strength is to allow a useful structure to emerge from the distributed interactions of the nodes taking into account that individual decisions represent a trade-off between the Euclidean distance (cost) and the geodesic distance (benefit).

## Methods

**Figure 1:**
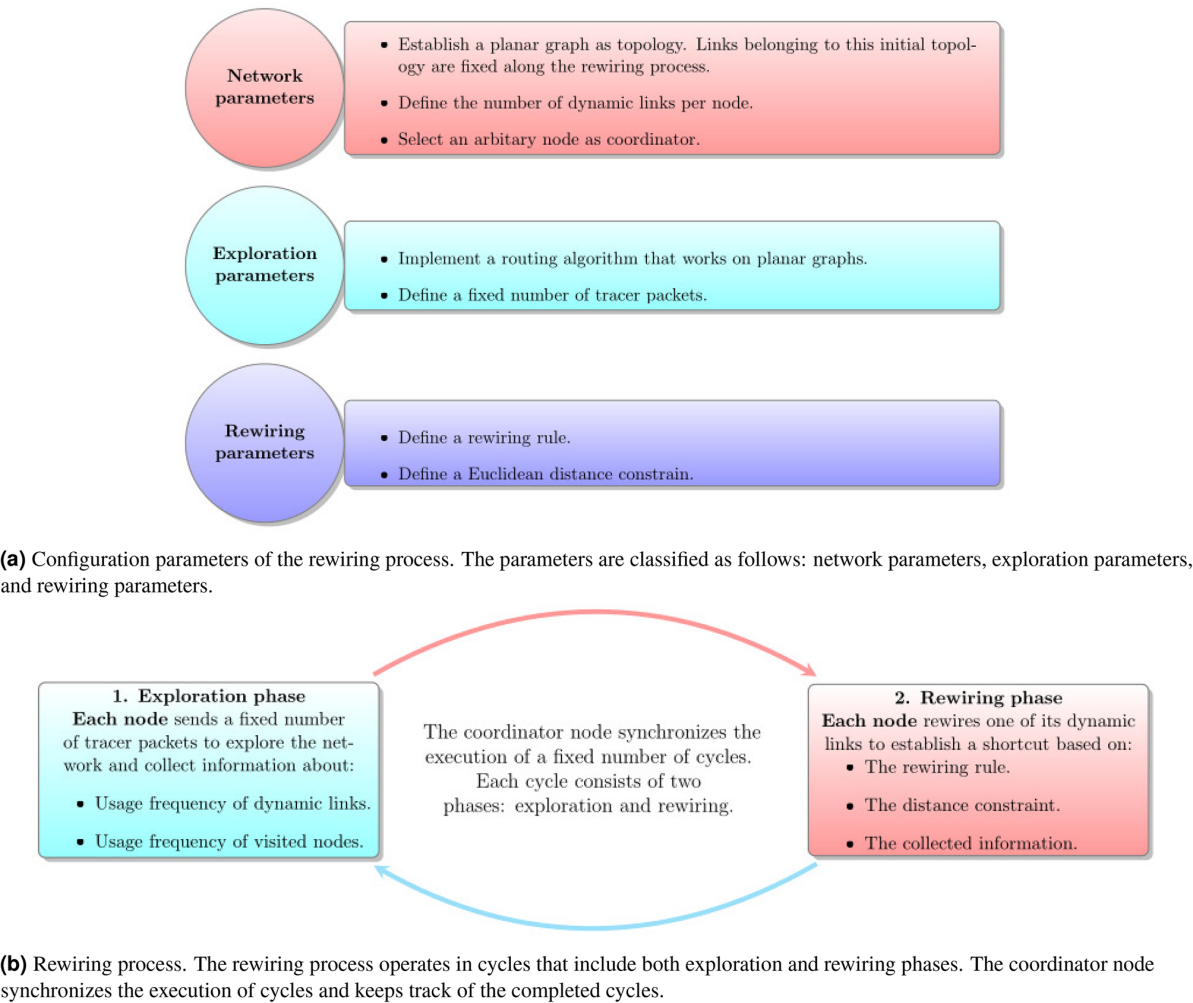
Overview of the distributed geometric rewiring model.

The proposed rewiring process allows turning networks whose underlying topology is given by a planar graph into complex networks. In the initial topology, in addition to fixed links, nodes have a given number of dynamic links that can be rewired. The rewiring process is executed through cycles in a synchronous and distributed manner. Each cycle consists of two phases: exploration and rewiring. During the former, each node exchanges packets to random chosen destinations, to acquire partial information about the current network topology. Meanwhile, during the latter, nodes use the collected information to make rewiring decisions that allow them to establish shortcuts that satisfy a Euclidean distance constraint. Figure 1 presents an overview of the rewiring process. Before explaining the rewiring process in detail, it is necessary to give a formal definition of the initial topology and the routing algorithm used to explore the network.

### Initial topology

Since the rewiring process was originally designed to work on infrastructure networks^17^, like telecommunication networks, the length of physical links and the Euclidean distance between nodes are restrictions that must be taken into account. Thus the initial topology is represented by a planar graph, which is embedded into the Euclidean space. Each node is then labeled with coordinates corresponding to its position in this space. Links belonging to this initial topology are fixed along the rewiring process. In addition to its fixed links, each node has a given number of dynamic links that are initially only connected to their owner nodes, and can be understood as loose ends. The loose end of each dynamic link is rewired along the process, while the end connected to its owner remains fixed. The maximum length that a dynamic link can be stretched is given in terms of a Euclidean distance, it can be understood as the limit on the amount of resources that a node may allocate in order to build geodesic shortcuts.

### Compass routing

The rewiring process requires a routing algorithm to explore the network and to exchange information between nodes. We propose to use the Compass Routing algorithm, which was designed to work on telecommunication networks whose only restriction is that they are planar networks^18^. Compass routing ensures packet delivery in planar graphs. However, it does not offer guarantees in some outerplanar graphs.

**Figure 2:**
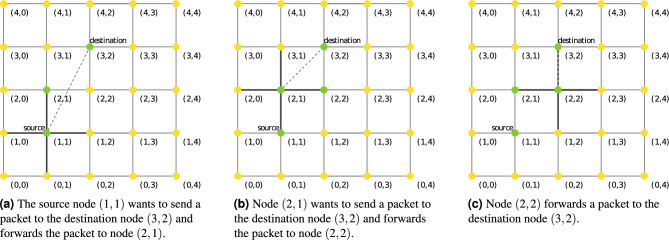
An example of Compass Routing on the $5\times 5$ grid graph, where nodes have been labeled with a coordinate in the Euclidean space. The source node (1, 1) wants to send a packet to destination node (3, 2). (**a**) First, straight lines are drawn from the source node to its neighbors ((0, 1), (1, 0), (1, 2) and (2, 1)) and to the destination node, then the packet is send to the node with the smallest angle between its line and the destination node line, i.e., (2, 1). (**b**) Then, the process is repeated in the node (2, 1), where nodes (2, 2) and (3, 1) have the smallest angle ($45^\circ$) between their lines and the destination node line, the packet is send to node (2, 2). (**c**) Finally, node (2, 2) sends the packet to the destination node, because the angle between its line and the destination node line is $0^\circ$.

 In this algorithm, the initial network is embedded into the Euclidean space and nodes are identified with coordinates in this metric space. The procedure for forwarding packets between two nodes starts at the source node by drawing straight lines from the source node to its neighbours and to the destination node. All these lines intersect each other at different angles. Data packets are forwarded to the adjacent node with the smallest angle between its line and the destination node line. Ties are broken randomly. The same procedure is repeated in the previously selected node until each routed packet reaches its destination node. Figure 2 shows an example of compass routing on the $5\times 5$ grid graph.

### Rewiring process

It is worth mentioning the difference between the rewiring mechanism and the rewiring process. The mechanism refers to the rules that each node follows during the process. In due time, the process produces different results on each node, depending on its local conditions that are different for each case. The rewiring process is executed through cycles in a synchronous and distributed manner. As was mentioned above, each cycle consists of two phases: the exploration phase that allows nodes to acquire partial information of the network topology and the rewiring phase, where nodes make rewiring decisions based on the collected information and subject to a constraint on the length of their dynamic links. We consider that the length of dynamic links is given by the Euclidean distance between their incident nodes. To allow the nodes explore the network in the same conditions, it is necessary to synchronize the beginning of each phase (and cycle). Therefore, at the beginning of the rewiring process, an arbitrary node is designated as coordinator. This node is in charge of the following tasks: to synchronize the beginning of exploration and rewiring phases, to keep a counter of the number of cycles that have been executed, and to finish the rewiring process when the desired number of cycles has been executed. The rewiring process begins when the coordinator starts a Propagation of Information (PI) algorithm^19^ to spread packets notifying nodes to start the exploration phase. These packets also allow the coordinator to notify its coordinates to the rest of the nodes.

#### Exploration phase

**Figure 3:**
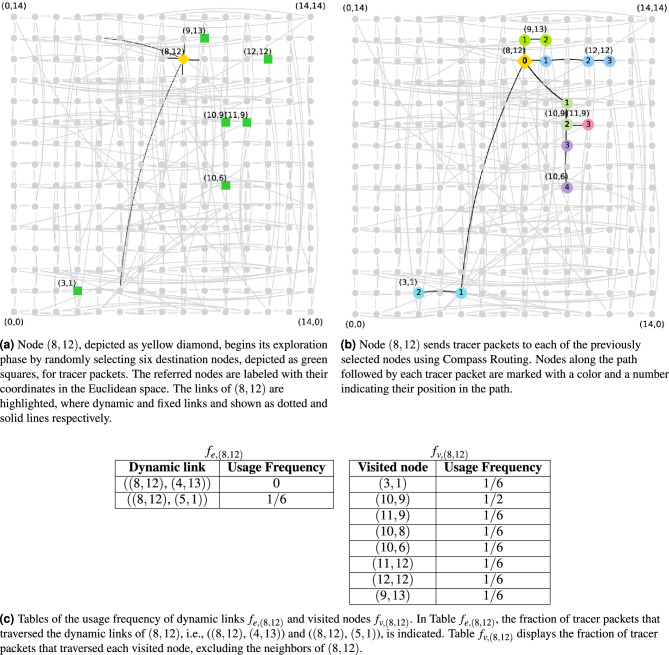
An example of the exploration phase on a rewired network obtained after 10 cycles of the rewiring process executed on an initial topology given by the $15\times 15$ grid graph. Nodes are placed and labeled according to their locations in the original grid graph, which is embedded into the Euclidean space. The node executing its exploration phase is (8, 12) and the number of tracer packets is set to 6. (**a**) At the beginning of its exploration phase, node (8, 12) randomly selects six nodes as destination for tracer packets. (**b**) Then, node (8, 12) sends a tracer packet to each of the previously selected nodes using Compass Routing. (**c**) Finally, tables of usage frequency of (8, 12), i.e., $f_{e,(8,12)}$ and $f_{v,(8,12)}$, are filled with the information about the paths followed by tracer packets.

A node starts its exploration phase when it receives a notification from the coordinator node. During this phase, each node sequentially sends a given number of tracer packets to random chosen destination nodes. Tracer packets are routed applying Compass Routing. Destination nodes respond to a tracer packet with an acknowledgment packet containing the path followed by the tracer packet. Each acknowledgment packet is routed backwards, through the path followed by its corresponding tracer packet. When a source node *i* receives an acknowledgment packet, it stores information about the usage frequency of its dynamic links and the frequency of the nodes whose tracers have visited. This information is stored by node *i* in the following tables:

- **Table of usage frequency of dynamic links** $f_{e,i}$. This table has two columns: dynamic link and usage frequency. Each entry of $f_{e,i}$ indicates the fraction of tracer packets that node *i* sent through one of its dynamic link, during the current cycle.
- **Table of usage frequency of visited nodes** $f_{v,i}$. This table has two columns: visited node and usage frequency. Each entry of $f_{v,i}$ indicates the fraction of tracer packets that node *i* sent and visited a node during the current cycle. The visiting frequencies to the neighbors of *i* are not stored.

A node finishes its exploration phase when it has received acknowledgment for each of its tracer packets. Then the node notifies the coordinator that it has finished its exploration phase. Figure 3 shows an example of the exploration for a rewired network whose initial topology is given by the $15\times 15$ grid graph. The exploration phase finishes when the coordinator is aware of the completion of this task at each node of the network, including itself. Then the coordinator node starts a PI algorithm to spread packets notifying nodes to start the rewiring phase.

#### Rewiring phase

A node *i* starts its rewiring phase when it receives a notification from the coordinator node. Then the notified node *i* makes a rewiring decision based on a rewiring rule and a constraint on the maximum length of its dynamic links $\ell _{\max }$, where the length of a dynamic link is given by the Euclidean distance *d*(*i*, *j*) between node *i* and the selected node *j*. Both the rewiring rule and $\ell _{\max }$ are given as parameters of the rewiring process. In this research work, we propose 2 rewiring rules that will be denoted as R1 and R2 respectively:

- **Rewiring rule 1 (**R1**)**. Node *i* rewires its least used dynamic link (according to table $f_{e,i}$) to the most visited node *j* (according to table $f_{v,i}$), if $d(i,j)\le \ell _{\max }$.
- **Rewiring rule 2 (**R2**)**. Node *i* rewires its least used dynamic link (according to table $f_{e,i}$) to a node *j* randomly selected from nodes in table $f_{v,i}$ that is at (geodesic) distance 2 from *i*, if $d(i,j)\le \ell _{\max }$.

Note that a node *i* can only rewire at most one dynamic link per cycle, however, if the selected node *j* does not satisfy the constraint $d(i,j)\le \ell _{\max }$, then node *i* will not rewire any of its dynamic links during this cycle. It is also important to mention that R1 and R2 introduce changes in the network topology at different speeds. Rule R1 introduces the fastest changes as it allows nodes to establish shortcuts with any node in the network. Meanwhile, R2 introduces the slowest changes as it restricts nodes to establish shortcuts in a very conservative manner, only with nodes at a geodesic distance of 2.

Returning to the example presented in Fig. 3, let’s analyze the rewiring decision of node (8, 12) under two cases with different rewiring rules and constraint values $\ell _{max}$. First, recall that the value of $\ell _{max}$ is given in terms of *L*, where $L\approx 19.79$ in the $15\times 15$ grid graph. Also, note that the least used dynamic link of node (8, 12) is ((8, 12), (4, 13)) according with $f_{e,(8,12)}$ (Fig. 3c).

- **Case i**: Consider the rewiring rule R1 and $\ell _{max}=L/8$, i.e., $\ell _{max}\approx 2.4$. Node (8, 12) does not rewire any of its dynamic links because the most visited node is at a Euclidean distance longer than 2.4. According to $f_{v,(8,12)}$ (Fig. 3c), the most visited node is (10, 9), which is at a Euclidean distance 3.6 from (8, 12).
- **Case ii**: Consider the rewiring rule R2 and $\ell _{max}=L/4$, i.e., $\ell _{max}\approx 4.9$. Node (8, 12) rewires its dynamic link ((8, 12), (4, 13)) to a randomly selected node from $f_{v,(8,12)}$, which is at a geodesic distance 2 from (8, 12), and at a Euclidean distance of at most 4.9 from (8, 12). The nodes satisfying such conditions are: (10, 9), (11, 12), and (9, 13). Although node (3, 1) is at a geodesic distance of 2 from (8, 12), it cannot be selected because its Euclidean distance from (8, 12) is 11.7, which is longer than 4.9.

A node finishes its rewiring phase when it has performed a rewiring decision, after which it must delete the current cycle information stored in its tables $f_{e,i}$ and $f_{v,i}$. Then the node notifies the coordinator that it has finished this phase. The rewiring phase of a cycle finishes when the coordinator node has finished its own rewiring phase and it has been notified that all the nodes in the network also have finished their rewiring phase. Then the coordinator node updates the cycle counter and checks if all cycles have been completed. If so, the coordinator node starts a PI algorithm to spread packets notifying nodes that the rewiring process has finished. Otherwise, the coordinator node starts a PI algorithm to spread packets notifying nodes to start the exploration phase of a new cycle.

### Communication cost and time complexity

Let us recall that each cycle through which the network evolves consists of 2 phases: exploration and rewiring, each controlled by an underlying synchronization stage. During the exploration phase of cycle *k*, each node issues *c* tracer packets, and each packet travels a maximum (geodesic) distance equal to the current network diameter $d_k$. Then the total number of packets exchanged during the exploration phase of cycle *k* will be at most $n \cdot c \cdot d_k$, where *n* denotes the order of the network. i.e., the number of nodes in the network. Meanwhile, during the rewiring phase, each node establishes a new shortcut, which involves the exchange of two packets to confirm the new connection between the issuing node and the node chosen as the shortcut. Then, the total number of packets exchanged during the rewiring phase of cycle *k* will be at most $2\cdot n\cdot d_k$. Turning now to the time complexity, let us assume that the transmission time of a packet is proportional to the geodesic distance it travels. Then each phase takes $O(d_k)$ time units. Finally, each exploration and rewiring phase is synchronized using a PI algorithm whose communication cost and time complexity are *O*(*m*) and $O(d_k)$, respectively, where *m* is the size of the network, i.e. the number of links in the network^19^.

**Table 1:** Communication cost and time complexity of a rewiring cycle.

	Communication cost	Time complexity
Exploration phase	$O(n\cdot d_k)$	$O(d_k)$
Rewiring phase	$O(n\cdot d_k)$	$O(d_k)$
Synchronization stage	*O*(*m*)	$O(d_k)$
Cycle *k*	$O(m+n\cdot d_k)$	$O(d_k)$

Table 1 summarizes the communication cost and time complexity of a cycle of the rewiring process. As we can see, the time complexity depends on the network diameter $d_k$ of the current cycle *k*, which is reduced at each cycle. It implies that on each new cycle the algorithm takes less resources than those involved during the previous round. However, this reduction depends on the rewiring rule (either R1 or R2) and the distance constraint $\ell _{\text {max}}$. Preliminary empirical evidence suggests that for an initial grid graph of order *n* and diameter *d*, the network reshapes itself to achieve a final diameter equal to $O(\log (n))$^17^.

## Results

In this section, we present topology and robustness analyses for a set of networks resulting from the proposed rewiring model. We have defined 12 different configurations of the rewiring process, which are described in the following subsection, then we have executed 10 experiments for each configuration. From each of these configurations results a network, then we have 10 networks for each configuration.

### Configuration of the rewiring process

We proposed the $50\times 50$, where each node of this initial grid graph was provided with two extra dynamic links. Although the rewiring process was designed to function with any planar graph as the initial topology, we choose the $50\times 50$ grid graph as the initial topology. Grid graphs possess an isometric embedding into the Euclidean space, rendering them a suitable framework to study distributed processes in networks embedded into the Euclidean space^20,21^.

The rewiring process was configured to run for 30 cycles. During the exploration phase of a cycle, each node sends 20 tracer packets. The experiments were executed for both rewiring rules: R1 and R2 and six values of $\ell _{\max }$: *L*, *L*/2, *L*/4, *L*/16 and *L*/32; where *L* is the Euclidean distance between two nodes in the opposite corners of the $50\times 50$ grid graph, i.e., $L\approx 69.29$. Then we have 12 different configurations.

### Topology analysis

This analysis focuses in the following topology properties: degree distribution, entropy, distance metrics, clustering coefficient and community structure. The results presented in this section correspond to the mean values and standard deviations of the topological metrics analyzed.

#### Static network properties

Note that the number of nodes *n* and links *m* remains constant throughout the rewiring process, where $n=2500$ corresponds to the number of nodes in the 50Ã-50 grid graph, and $m=9900$ denotes the sum of 4900 fixed links in the 50Ã-50 grid graph along with 5000 dynamic links. Then the average node degree $\langle k \rangle$ and density *D* remain fixed, i.e., $\langle k \rangle =\frac{2m}{n}=7.92$ and $D =\frac{2m}{n(n-1)}=0.0031$.

**Figure 4:**
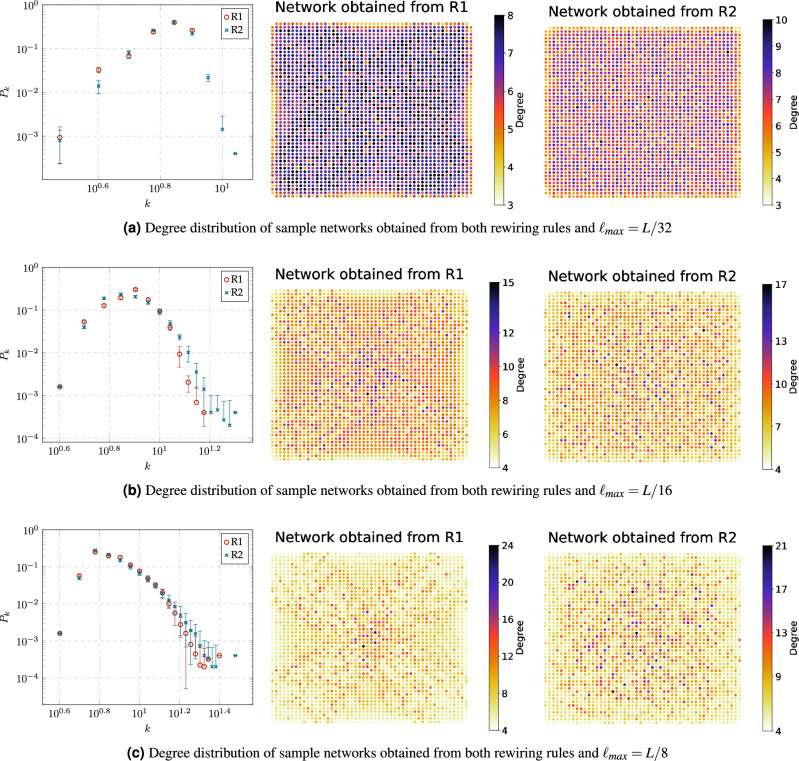
Degree distribution $P_k$ of sample networks obtained from both rewiring rules: R1 and R2, with constraint values $\ell _{max}=L/32$, $\ell _{max}=L/16$ and $\ell _{max}=L/8$. Nodes are placed according to their position at the original grid graph.

#### Degree distributions and entropy

Figure 4 shows the degree distributions of sample networks obtained from the rewiring rules R1 and R2 with constraint values $\ell _{max}=L/32$, $\ell _{max}=L/16$ and $\ell _{max}=L/8$. We notice that the degree distribution is similar to a negatively skewed binomial distribution when $\ell _{max}=L/32$ (Fig. 4a) and a positively skewed when $\ell _{max}=L/16$ (Fig. 4b). We conjecture that this effect can be explained by examining the likelihood of nodes capturing new dynamic links in our rewiring model (when $\ell _{max}=L/32$ and $\ell _{max}=L/32$) as the probability of success *p* in the binomial distribution

1 $$\begin{aligned} P_k \approx \left( {\begin{array}{c}n-1\\ k\end{array}}\right) p^k (1-p)^{n-1-k}. \end{aligned}$$

First, note that for a node *i* to capture a new link, it competes with others within a Euclidean distance of at most $\ell _{max}$ from it, excluding its neighbors. Let $n_{(i,\ell _{max})}$ denote the number of such nodes. Since each node has two dynamic links, the likelihood that node *i* captures a new link is $p=2/n_{(i,\ell _{max})}$, representing the probability of success *p* in Eq. (1). Consequently, if $\ell _{max}=L/32$, then $p\ge 0.5$ because $2\le n_{(i,L/32)}\le 4$ by the construction of the grid graph. On the other hand, if $\ell _{max}=L/16$, then $12\le n_{(i,L/32)}\le 36$ and $1/18\le p\le 1/6$. Also, when $\ell _{max}=L/32$, we observe that the degree distribution of R2 exhibits a more bell-shaped curve compared to R1 (Fig. 4a). This behavior could be attributed to several nodes selected by R1 do not satisfy the distance constrain $\ell _{max}$. In contrast, all nodes selected by R2 satisfy the constrain distance, and as a result, in R2, the probability of capturing a new node is normally distributed.

Turning now to the degree distribution resulting from the distance constrain $\ell _{max} = L/8$ (Fig. 4c), hubs begin to emerge because the increase in the number of nodes competing to capture a link, together with the rewiring rules, favors the phenomenon of preferential attachment. In the case of R1, hubs emerge in the center of the original grid graph because their initial position gives them an advantage for capturing new nodes. In the case of R2, hubs are distributed in the network because this rule restricts nodes to establish connections with others at a geodesic distance of 2.

**Figure 5:**
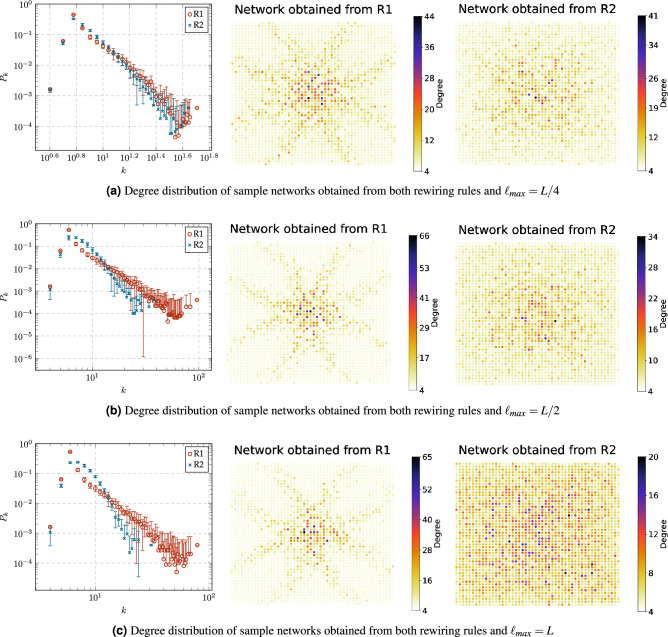
Degree distribution $P_k$ of sample networks obtained from both rewiring rules: R1 and R2, with constraint values $\ell _{max}=L/4$, $\ell _{max}=L/2$ and $\ell _{max}=L$. Nodes are placed according to their position at the original grid graph.

In the case of degree distribution resulting from distance constrains $\ell _{max} \ge L/4$, Fig. 5 shows that the preferential attachment phenomenon gains force as $\ell _{max}$ increases. In the case of R1, it is clear that the degree distribution follows a power law

2 $$\begin{aligned} P_k\approx k^{-\gamma } \end{aligned}$$

when $\ell _{max} \ge L/4$ (Fig. 5a). Additionally, the networks obtained from R1 have similar degree distributions when $\ell _{max} = L/2$ (Fig. 5b) and $\ell _{max} = L$ (Fig. 5c) because the majority of nodes selected by this rule satisfy both distance constraints. In the case of R2, when $\ell _{max} \ge L/4$, a hub emerges for each cluster of nodes that satisfy both R2 and the distance constraint. As a result, the number of hubs increases (and the maximum node degree decreases) as $\ell _{max}$ increases.

**Figure 6:**
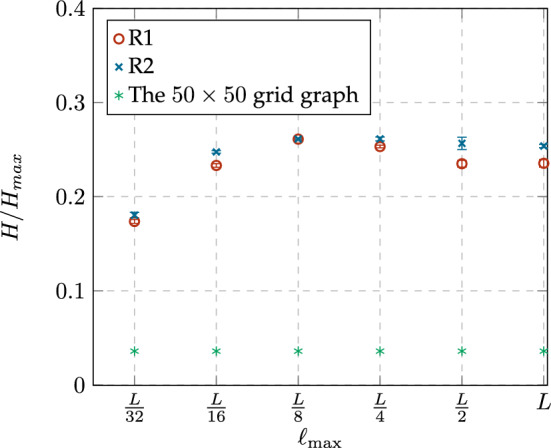
Comparison of the normalized Shannon entropy ($H/H_{max}$) of the original grid graph and the networks obtained from both rewiring rules, R1 and R2, with different constraint values $\ell _{\max }$.

Another important insight into the degree distribution is its utility in measuring the entropy structure of a network, indicating its structural complexity^22^. We employ the normalized Shannon entropy^23^ given by

3 $$\begin{aligned} H= -\sum _{k=0}^{n-1}p_k\log _2(p_k), \end{aligned}$$

which allows to measure the degree heterogeneity of a network. The maximum value of *H* is given for networks whose topology is a complete graph, then $H_{max}=\log _2(n-1)$, which can be used as the denominator for normalizing Eq. (3). Conversely, the minimum value of *H* is given for networks whose topology is a star graph, then $H_{min}=0$. Figure 6 shows a comparison between the normalized Shannon entropy, denoted as $H/H_{max}$, of the original $50\times 50$ grid graph and the networks obtained from the rewiring rules R1 and R2 with different constraint values $\ell _{\max }$. In both cases, R1 and R2, the values of *H* follow very similar trends: *H* increases from $\ell _{max} = L/32$ to $\ell _{max}=L/8$, when the degree distributions are similar to binomial distributions. On the other hand, *H* slowly decreases from $\ell _{max}= L/4$ to $\ell _{max}=L$ as the preferential attachment phenomenon arises. The maximum value of *H* is achieved when $\ell _{max}=L/8$, coinciding with the onset of hub formation.

#### Distance metrics

**Figure 7:**
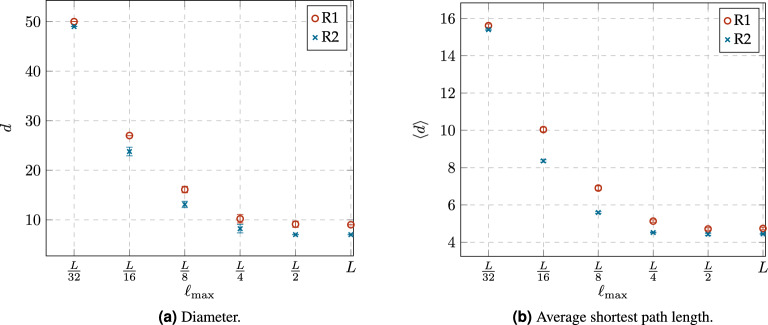
Comparison of diameter *d* and average shortest path length $\langle d\rangle$ of networks obtained from both rewiring rules, R1 and R2, with different constraint values $\ell _{\max }$.

The basic distance metrics between nodes in a network are the diameter *d*, which is given by the length of the maximum shortest path in the network, and the average shortest path length $\langle d \rangle$. Figure 7 shows a comparison between the values of *d* and $\langle d\rangle$ of networks obtained from the rewiring rules R1 and R2 with different constraint values $\ell _{\max }$. We observe that both rules produce very similar results, i.e., both achieve a rather short *d* and $\langle d \rangle$, mainly when the constraint value $\ell _{max}$ provides enough *“stretching”* to build effective shortcuts on the initial graph. We conjecture that the slight difference between the rewiring rules can be explained from the way each rewiring rule selects the candidate node to which it aims its next dynamic connection. In R1, each node identifies a common target and every effort is oriented to settle a link aiming to this position. Then nodes in R1 tend to reinforce the construction of a limited number of hierarchical paths. In contrast, we could say that R2 is a blind search that selects an arbitrary node at distance 2. Then nodes in R2 explore the construction of a wider set of paths which in turn include shorter solutions.

#### Average clustering coefficient

The local clustering coefficient of a node *i*, denoted by $C_i$, determines the proportion of edges between the $k_i$ neighbors of *i* with respect to the edges in the complete graph with $k_i$ nodes, i.e., $K_{k_i}$. Let $E_i$ be the number of edges between the $k_i$ neighbors of *i*, then $C_i$ is given by

4 $$\begin{aligned} C_i=\frac{2E_i}{k_i(k_i-1)}. \end{aligned}$$

The value of $C_i$ can be interpreted as a measure of the local density of the neighborhood of a node *i*, and in terms of probability, it represents the probability that two randomly selected neighbors of *i* are connected. We have analyzed the average clustering coefficient $\langle C\rangle$, which is a global measure of the degree of clustering in a network and is given by

5 $$\begin{aligned} \langle C\rangle =\frac{1}{n}\sum _{n}^{i=1}C_i. \end{aligned}$$

**Figure 8:**
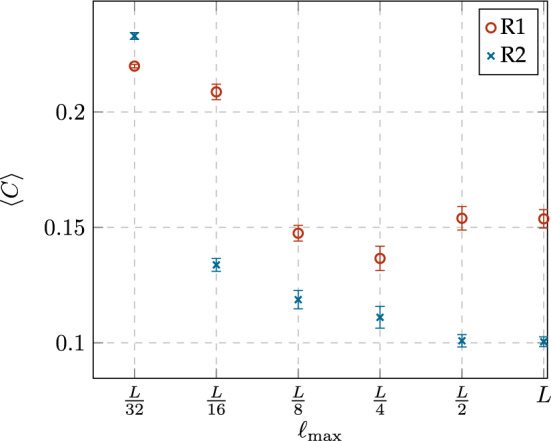
Comparison of the average clustering coefficient $\langle C\rangle$ of networks obtained from both rewiring rules, R1 and R2, with different constraint values $\ell _{\max }$.

Figure 8 shows a comparison between the values $\langle C\rangle$ of networks obtained from the rewiring rules R1 and R2 with different constraint values $\ell _{\max }$. The first observation that we draw from this figure is that the value of $\ell _{\max }$ does not have a strong impact on $\langle C\rangle$ except for the case of the shortest dynamic links, i.e., $\ell _{\max }= D / 32$. Under these circumstances, it seems that both rewiring rules behave in a very similar way and produce the highest $\langle C\rangle$ . This behavior can be explained from the fact that, when a node has a link which *“stretches”* the least possible, the number of nodes to connect is rather small and this selection reinforces the construction of a tighter community. Instead, for longer links, rewiring rules have the strongest influence on the resulting measure. On the other hand, the slight difference between the rewiring rules can be explained, as we mentioned above, based on the existence of a common target (R1), or the lack of it (R2). From these different goals we conclude that R1 fosters the construction of a central community (or communities) whose nodes aim to i) either a central position (of the original grid graph), or ii) a node of case i. Notice that this is a recursive definition that also explains the arising, not only of a stronger community, but also of a hierarchy. In contrast, under R2, communities are shaped by 2 *“forces”* not necessarily aligned: i) the closeness of the selected node, but ii) the lack of a common target. From our point of view, the second weighs more and it explains the lower $\langle C\rangle$, compared to R1.

#### Community structure

Community detection algorithms compute a network partition consisting of locally dense connected subgraphs called communities. We employed the Louvain algorithm^24^ to detect the community partition of the networks obtained from the rewiring rules R1 and R2 with different constraint values $\ell _{\max }$. The Louvain algorithm works by optimizing the modularity *M* of the community partition, which measures the sharpness of the underlying communities and is given by

6 $$\begin{aligned} M=\sum _{j=1}^{n_C}\left[ \frac{m_{C_j}}{m}-\left( \frac{k_{C_j}}{2m}\right) ^2\right] , \end{aligned}$$

where $n_C$ is the number of detected communities, $m_{C_j}$ is the number of edges in the community *j*, and $k_{C_j}$ is the sum of node degrees in the community *j*. A higher value of *M* indicates that communities are clearly defined and barely connected by a small set of *“cut edges”* among them; note that the maximum value of *M* is 1. In contrast, a modularity close to 0 suggests that communities are strongly intertwined and barely separated from each other. Another important measure of community partition quality is the ratio of edge cut $E_C$, indicating the proportion of edges or links that join the communities. These links do not belong to any community, then

7 $$\begin{aligned} {E_C=1-\frac{\sum _{j=1}^{n_C}m_{C_j}}{m}.} \end{aligned}$$

A smaller $E_C$ means that the communities are more cleanly separated. In contrast, a bigger value means that the borders between communities are rather fuzzy.

**Figure 9:**
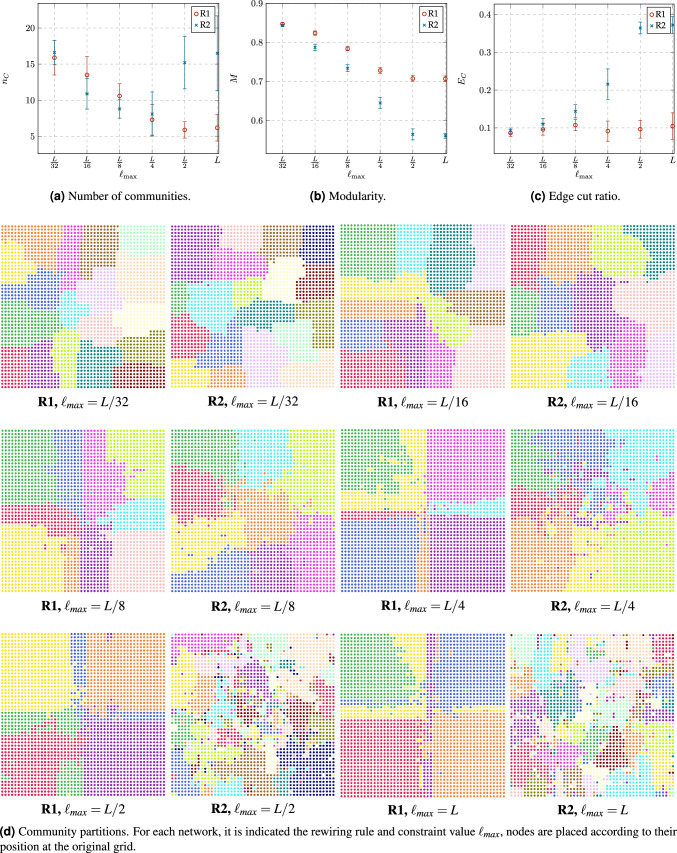
Comparison of community metrics: number of communities $n_C$, modularity *M* and edge cut ratio $E_C$; and community partitions of sample networks obtained from both rewiring rules: R1 and R2, with different constraint values $\ell _{max}$.

Figure 9 shows a comparison between the values of $n_C$, *M* and $E_C$, as well as samples of community partitions for networks obtained from both rewiring rules with different constraint values $\ell _{max}$. Before explain in detail this figure, it is worth noting from Fig. 9d that both rewiring rules with low values of distance constraint, i.e., $\ell _{max}\le L/4$, result in a community partition of several local communities with high quality (high *M*) and simple boundaries (low $E_C$). However, each rewiring rule with $\ell _{max}=L/2$ and $\ell _{max}=L$ gives very similar results. In the case of R1, a low number of communities with acceptable quality emerge, while in the case of R2, several communities with low quality (low *M*) and complex boundaries (high $E_C$) emerge.

To explain this results, we have to recall that the order of the network, i.e., *n*, is fixed. Therefore, a higher value of $n_C$, (Fig. 9a) implies that the order of each community is, on average, rather smaller compared to the case of a smaller number of communities. Figure 9a supports the conjecture that we made when analyzed the distance metrics, that is to say: nodes in R1 strengthen the communities around the hierarchical hubs. Therefore (considering $\ell _{\max }\ge L/4$ ), $n_C$ in R1 is significantly less than R2 where each node has a wider set of options to connect its dynamic links. In other words, R1 achieves a smaller number of communities, each with a higher order, compared to the resulting communities developed by R2. On the other hand, as we have stated, nodes using R2 explore more options. But, when their dynamic link is shorter, i.e., $\ell _{\max }\le L/8$, this rule reinforces the possibility of strengthening slightly bigger communities (compared to R1). Meanwhile, a node that uses R1 bets on strengthening the few local hubs that lie in the initial neighborhoods, which means that R1 contributes to the emergence of a bigger number of (smaller) communities. In the limit case ($\ell _{\max }=L/32$) the behavior of both rules is indistinguishable from each other, as the possibilities to connect the dynamic links collapse to a minimal set.

Figure 9b shows that, as we have mentioned before, (i) when the length of the dynamic link is very short, communities achieve the same properties regardless of the rewiring rule that builds them. In contrast, (ii) when the link length offers a longer leeway, rewiring rules weigh the most on the resulting properties. Also, in the first case (i) communities show sharper borders, since nodes can only reinforce their initial neighborhoods. Meanwhile, in case (ii) as $\ell _{max}$ increases, borders start blurring. Nevertheless, the impact of the rewiring rule is more pronounced in this last case: nodes using R1 set their connections around the hubs that make up the hierarchy and this reinforces the sharpness of the resulting communities. In contrast, nodes using R2 do not have a common set of targets and this induces the construction of a bigger number of *“cut edges”* between the otherwise separated communities. To reinforce these conclusions, it is worth considering the value of $E_C$ in Fig. 9c. In the case of R1, communities are more cleanly separated, resulting in small values for $E_C$. Conversely, in the case of R2, the borders between communities are rather fuzzy, leading to higher values for $E_C$. Also, note that when considering the possibility of a degradation process, such as an attack aiming to disconnect the overall network with the least possible effort, a higher value of *M* is not necessarily good news. We will revisit this issue in the last part of our study.

### Robustness analysis

Thus far, this study has focused on the topology analysis of networks resulting from the proposed rewiring model. The second part of the study presents a robustness analysis for these networks under degradation processes, including attacks and failures scenarios.

#### Attacks and failures scenarios

To evaluate the robustness of networks resulting from our rewiring model, we consider the sequential elimination of nodes under attack and failure scenarios. In the case of attacks, the highest degree node is selected at each simulated attack, while in the case of failures, nodes are randomly chosen one after the other. To obtain accurate results, in the case of failures, we executed 10 experiments for each network. In both scenarios, we estimate network robustness by recording a set of connectivity and communication measures that demonstrate the progressive degradation of the analyzed networks as a function of the fraction of removed nodes *f*^25^.

#### Connectivity robustness

Network connectivity means that there is at least one path between any pair of nodes. The prevailing metric for assessing connectivity robustness in networks is

8 $$\begin{aligned} R=\frac{1}{n} \sum _{p=0}^{n-1}N_{LCC}(f)=\frac{1}{n}\sum _{p=0}^{n-1}\frac{n_{LCC}(p)}{n-p}, \end{aligned}$$

where *n* denotes the order of the original network, while $n_{LCC}$ and $N_{LCC}$ respectively represent the order and relative order of the largest connected component (LCC) after a fraction $f=p/n$ of nodes have been removed from the network. Although $N_{LCC}$ is also known as *“the fractional size of the LCC”*^26^, in Graph Theory, *“size”* refers to the number of edges in a given graph, while *“order”* refers to the number of nodes^27^, which is what $N_{LCC}$ represents. Therefore, we refer to the order of the LCC instead of the size of the LCC^25,28^. Note that $N_{LCC}$ is measured with respect to the remaining nodes in the network, i.e., $n-p$, as we consider that *p* malfunctioned nodes are removed from the network. In another approach, some authors consider the malfunctioned nodes as still being part of the network, and they measure $N_{LCC}$ with respect to the order of the original network, i.e., *n*^26,28^. The range of possible values of *R* spans from 0 to 1, with these extremes representing the least robust networks (star networks) and the most robust networks (fully-connected networks), respectively.

In addition to connectivity robustness as defined by Eq. (8), the evolution of $N_{LCC}$ during a degradation process is a crucial aspect in the study of network robustness. A low value of $N_{LCC}$ indicates severe disturbance or malfunctioning of the network. Therefore, there exists a critical point in $N_{LCC}$ at which the network transitions from a functional state to a damaged state. It is well-known that this transition is rapid in many complex networks^25,26^. Thus, the function $N_{LCC}(f)$ remains nearly flat with values close to 1 until the critical area, where the network rapidly collapses, and $N_{LCC}(f)$ takes values close to 0. Under such conditions Eq. (8) can be approximated by the Mean Value Theorem^29^ to $f^*$ such that $N_{LCC}(f^*)\approx 0.5$. Since $N_{LCC}(f^*)$ represents the mean value at which the network collapses, we define the critical point $f^*$ as follows:

9 $$\begin{aligned} N_{LCC}(f^*)\approx 0.5. \end{aligned}$$

Although $R \approx f^*$ under the conditions explained above, both metrics have different meanings. While *R* indicates the degree of connectivity robustness, $f^*$ indicates the fraction of nodes that must be removed to transition the network from a functional state to a damaged state. To estimate the velocity of this transition, we also define the critical area as the percentage of removed nodes *f*, such that

10 $$\begin{aligned} 0.1\le N_{LCC}(f)\le 0.9. \end{aligned}$$

**Figure 10:**
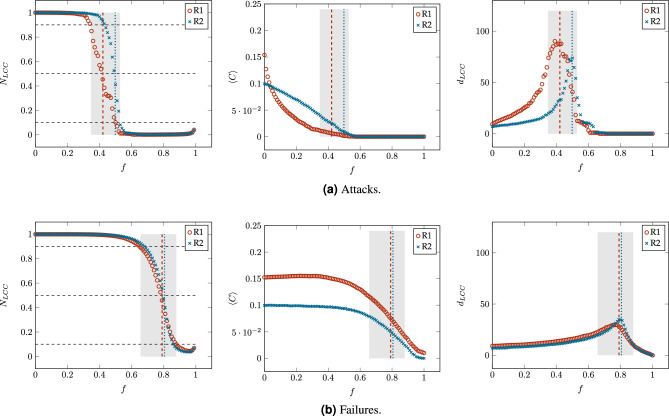
Changes in the relative order of the Largest Connected Component ($N_{LCC}$), the average clustering coefficient ($\langle C\rangle$), and the LCC diameter ($d_{LCC}$) as a function of the fraction of removed nodes *f* in the networks obtained from both rewiring rules, R1 and R2, with a constraint value of $\ell _{\max }=L/2$. The shaded sections corresponds to the critical area, where $0.1\le N_L(f)\le 0.9$, while the vertical lines indicate the critical point $f^*$, where dashed and dotted lines corresponding to networks resulting from rules R1 and R2, respectively.

Figure 10 shows an example for the changes in the order of the LCC ($N_{LCC}$), the average clustering coefficient ($\langle C\rangle$) and the diameter of the LCC ($d_{LCC}$) as function of the proportion of removed nodes (*f*) for the networks obtained from R1 and R2 with the constraint value $\ell _{max}\le L/2$. Figure 10a and b correspond to results under attacks and failures respectively. The leftmost figure on each case correspond to $N_{LCC}$ as a function of *f*, in both cases we identify the critical area (shaded), see Eq. (10), and the critical point $f^*$ (vertical line), see Eq. (9). In both cases, the value of $N_{LCC}$ starts from an initial value equal to 1 and decreases rapidly through 3 different stages: (1) a slow decline, (2) a rather rapid collapse (critical area), and (3) a final steady condition where the largest component includes only a few of the remaining nodes. Differences on the results arise from 2 main causes: either the nature of the degradation scenario (attacks or failures), or the nature of the network itself (networks built from either rule R1, or R2). The central and the rightmost figure, in either Fig. 10a or Fig. 10b , correspond to $\langle C\rangle$ and $d_{LCC}$, as functions of *f*, respectively. We also marked the critical area and critical point as defined in Eq. (10) and Eq.(9) respectively. Regarding $N_{LCC}$, upon exiting the critical area, in both cases (R1 or R2), the network has less than $20\%$ of the original nodes, and of these, less than $10\%$ belong to the LCC (see Fig. 10 on the leftmost, or $N_{LCC}$ as a function of *f*). In the case of $\langle C\rangle$, under attacks, its value falls to 0, after $f^*$ is reached. Meanwhile, under failures, after the critical area, $\langle C\rangle$ does not reaches the value of zero immediately, but it drops rapidly. Finally, regarding the changes in $d_{LCC}$, it appears to grow because up until before $f^*$, the LCC has $50\%$ of the surviving nodes, and up to this point, nodes removal only caused shortcuts to be lost, thus increasing the diameter. However, after $f^*$, nodes removal rapidly fragments the largest component, leading to a reduction in $d_{LCC}$.

**Figure 11:**
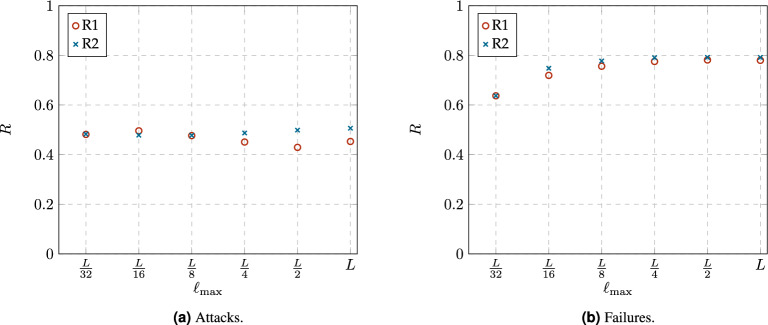
Comparison of the connectivity robustness *R* for the networks obtained from both rewiring rules, R1 and R2, with different constraint values $\ell _{max}$.

**Figure 12:**
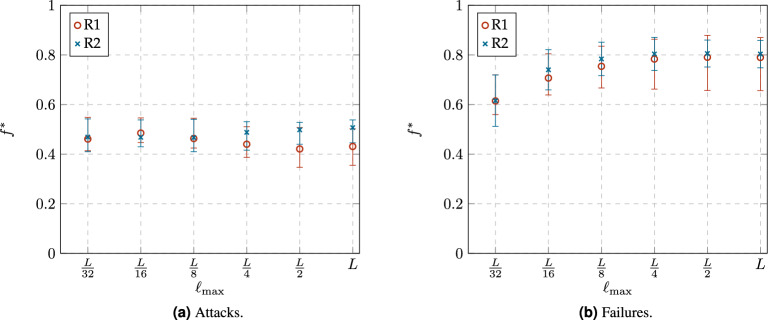
Comparison of the critical point $f^*$ for the networks obtained from both rewiring rules, R1 and R2, with different constraint values $\ell _{max}$. The error bars corresponds to the limits of the critical area, where $0.1\le N(f)\le 0.9$.

Figures 11 and 12 summarize the values of *R* and $f^*$, respectively, for the analyzed networks. The error bars in 12 corresponds to the limits of the critical areas (Eq. (10)), where the length of the critical area (error bar) conveys the idea of speed of degradation, which is also an inherent trait to the network under study. As expected, the values shown in both figures are very similar, which confirms the effectiveness of the proposed critical point $f^*$.

#### Communication robustness

Network communication refers to the reachability of any two nodes in the network to exchange information. An important measure of communication robustness is the Average Two-Terminal Reliability (*A*2*TR*) defined as the average probability that a randomly chosen pair of nodes is connected, and given by

11 $$\begin{aligned} A2TR( f)=\frac{\sum _{i=1}^{|CC|}{\left( {\begin{array}{c}n_{Ci}\\ 2\end{array}}\right) }}{\left( {\begin{array}{c}n-p\\ 2\end{array}}\right) } \end{aligned}$$

where *n* denotes the order of the original network, *p* denotes the number of removed nodes, |*CC*| denotes the number of connected components, and $n_{Ci}$ denotes the order of the *i*-th connected component, both after a fraction $f=p/n$ of nodes have been removed from the network^30^. The numerator of Eq. (11) represents the number of communicable pairs of nodes, while the denominator represents the number of all pairs of nodes in the network. If the network is connected, $A2TR=1$; otherwise $A2TR<1$.

The evolution of *A*2*TR* illustrates how a network, under a degradation process, breaks into components. Similar to the evolution of $N_{LCC}$, there exists a threshold in *A*2*TR* where the network under study transitions from a functional state to a damaged state. This threshold can be estimated by the metric called $\mu \text {-}A2TR$, which is defined as:

12 $$\begin{aligned} \mu \text {-}A2TR=\frac{\sum _{p=0}^{n-2}A2TR(f)}{n-1}, \end{aligned}$$

where *A*2*TR*(*f*) denotes the *A*2*TR* value of the network when a fraction of $f=p/n$ nodes have been removed^31^. As the critical point $f^*$ proposed in Eq. (9), $\mu \text {-}A2TR$ takes values between 0 and 1. The higher the value of $\mu \text {-}A2TR$, the more robust the network is in terms of connectivity, as it indicates that it is more difficult to break it into several clusters.

**Figure 13:**
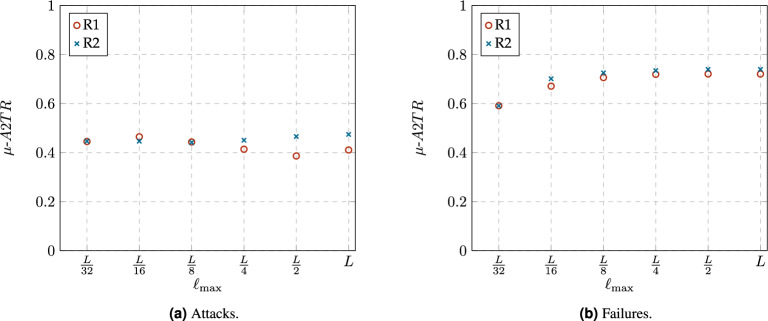
Comparison of $\mu$-*A*2*TR* for the networks obtained from both rewiring rules, R1 and R2, with different constraint values $\ell _{max}$.

Figure 13 summarizes the values of $\mu \text {-}A2TR$ for the analyzed networks. As the values of *R* in Fig. 11, the values of $\mu \text {-}A2TR$ are very similar to those obtained by our proposed $f^*$, see Fig 12, indicating that the networks obtained from our rewiring mechanism have similar connectivity and communication robustness.

We also consider that the exploration phase can be extended or enriched, allowing each node to have more elements to make decisions. For example, nodes could receive a report that includes information about the degree of the nodes visited, the availability of resources to handle external requests, the presence of contingencies in certain regions of space, etc. Additionally, the use of dynamic links could give rise to different intertwined or overlapping structures. For instance, one subgraph could consist of the shortest routes, while another subgraph contains the least congested routes, and so on. At some point in our work we conjectured that the routing algorithm used to explore the network did not have a greater incidence on the emergent network and the stabilization of topology properties during the rewiring. However, our preliminary results in this direction indicate otherwise. Likewise, the rules with which rewiring decisions are made deserve attention. We say that R1 makes a more informed decision, while R2 makes a “nearly” blind decision. However, there are situations in which networks resulting from R2 seems to offer better characteristics (for example, higher robustness). For its part, R1 can give rise to a super hub node with which everyone wants to connect. Perhaps, a rewiring rule could be investigated in which the recognized nodes are chosen, following some probability distribution. Another intriguing question is how the initial graph could be embedded in other non-Euclidean spaces, or what other types of non-planar graphs could be utilized as initial topologies. This consideration intersects with the selection of routing algorithms, as our current focus has been on a single algorithm, Compass Routing, which operates specifically on planar graphs.

Generalizing our proposal, we might envision a system of interconnected agents existing within a metric space, wherein they conduct a series of explorations or sampling procedures to determine how to allocate a portion of their resources. Their objective is to enhance access to various regions within the metric space. Although this exploration process is somewhat random and partial, it can rapidly give rise to different network properties that strike a balance between the objectives of individual agents (nodes) and the overall system (network). One emergent property could be the network’s ability to face various forms of degradation. Within this approach, agents might request services from others, but they could also act as service providers, thereby establishing a system where cooperation is intrinsic.

Finally, we believe that the emergence of complex structures under geometric constraints can provide insights into the effects of rewiring mechanisms under limited resources, a scenario that may arise in various contexts. From this perspective, we identify two application approaches. The first, which we have explored in this article, involves rewiring a network through a finite number of cycles to achieve a topology with specific desirable topological and robustness properties. The second approach involves modeling a *“living”* system as a network where there is not necessarily a limited number of execution cycles. This would enable us to describe agent systems, such as a network superimposed on a physical structure, e.g., a P2P network or a network on a chip. From this approach, we can define rules that allow the system to self-regulate, even under changing or unstable conditions. This possibility raises new questions, such as whether equilibrium states are reached, whether the system can enter unstable conditions, and how disruptive events are managed. Within the second approach, we can recognize different strategies that induce cooperation in various ways. Initially, we can identify a system consisting of nodes or agents that cooperate according to the proposed rewiring model. Additionally, heterogeneous strategies can be proposed, where strategies with varying levels of cooperation are considered.

## Conclusions

In this study, we introduced a novel distributed geometric rewiring model that initiates with a planar graph embedded into the Euclidean space. The nodes within this network represent active entities or agents, and the links between them denote potential interactions. Each node is equipped with two kinds of links: static and dynamic. Static links are inherent to the network’s initial topology, whereas dynamic links are utilized by nodes to form shortcuts to distant network regions through a series of exploration and rewiring cycles. During the exploration phase, nodes dispatch tracer packets to various destinations within the network. These packets, upon returning, provide crucial data for the decision-making process involved in the dynamic links’ rewiring. After several cycles, the network evolves into a structure exhibiting both new topological features—such as the preferential attachment phenomenon, small-world characteristics, and community structure—and enhanced functional attributes, like improved connectivity and robustness against targeted attacks and random failures. The synchronization of exploration and rewiring phases is vital for the accurate updating of network information, which can be managed either centrally by a single node or distributed across multiple nodes.

The model proposes two distinct rewiring rules: Rule R1 targets the most frequented node identified in the latest exploration phase, while Rule R2 chooses the first node encountered at a geodesic distance of two. Although both rules facilitate the emergence of network hubs, they differ in the speed at which these hubs form. Future research could explore alternative rewiring rules that either mitigate or deliberately influence hub formation. Moreover, we suggest enhancing the exploration phase to provide nodes with a richer dataset for decision-making. This could include information on node degrees, resource availability, and regional contingencies. The dynamic link mechanism might also enable the coexistence of multiple network structures, such as one prioritizing the shortest paths and another focusing on minimizing congestion. Preliminary findings challenge our initial assumption that the choice of routing algorithm has a minimal impact on the network’s emergent structure and stability. This underscores the importance of both the routing algorithm and the rewiring decision rules. Specifically, Rule R1 appears to make more informed decisions, while Rule R2’s approach, though less informed, can result in networks with superior robustness in certain scenarios.

An interesting avenue for future research is the exploration of network embeddings in non-Euclidean spaces and the use of non-planar graphs as initial topologies. This intersects with the selection of routing algorithms, emphasizing the need to diversify beyond our current focus on Compass Routing, which is tailored for planar graphs. Envisioning a broader application, our model suggests a system where interconnected agents undertake exploratory actions to find a trade-off between the cost of resource allocation (represented by the Euclidean distance), and the benefit to exchange information with the otherwise distant regions of the original structure (represented by the geodesic distance). This system, inherently cooperative, could adaptively respond to various forms of network degradation. In conclusion, our work sheds light on the potential of geometric constraints in rewiring mechanisms to foster complex network structures, even under resource limitations. We explored two primary applications: the finite-cycle rewiring of networks for desired topological and robustness traits, and the modeling of *“living”* networks capable of self-regulation and adaptation to dynamic conditions. This opens up new questions about equilibrium, system stability, and the management of disruptive events, offering a rich domain for further investigation into cooperative strategies and network dynamics.

## Data Availability

The dataset and code that support the findings of this study are available at https://doi.org/10.5281/zenodo.10799272. This is provided to facilitate reproducibility of the presented results.
